# Supplementary material for: Patterns of Intron Gain and Loss in Fungi
Source: PLoS Biol. 2004 Nov 30;2(12):e422. doi: 10.1371/journal.pbio.0020422 (PMC532390; doi:10.1371/journal.pbio.0020422)
Supplement: Table S1 — Also available at http://genes.mit.edu/NielsenEtAl/. (4.3 MB ZIP). [file pbio.0020422.st001.zip › NielsenEtAl/html/836.html]

AN9362.1.NCU03261.1.MG09948.1.FG06860.1


```
 CLUSTAL W (1.82) Multiple Sequence Alignments - Introns Inserted


Sequence 1: MG09948.1	209 aa
Sequence 2: FG06860.1	209 aa
Sequence 3: NCU03261.1	208 aa
Sequence 4: AN9362.1	209 aa
Alignment Length: 209 aa
Number Identitical Residues: 177 aa
Alignment Score (without introns) 7713


MG09948.1 	MANFLASIFGTEQDK0VNCSFYF~KIGACRHGDRCSRKHVKPTYSQTILMPNMYQNPAYD
NCU03261.1	MANFLASIFGTEQDK~VNCSFYY~KIGACRHGDRCSRKHVKPSYSQTILMPNLYHNPAFD
FG06860.1 	MANFLASIFGTELDK~VNCSFYF0KIGACRHGDRCSRKHVKPSYSQTILMPNLYQNPAYD
AN9362.1  	MANYLASIFGTEQDK~VNCSFYY~KIGACRHGDRCSRKHVKPSYSQTILMPNMYQNPAYD
          	***:******** ** ******: ******************:*********:*:***:*

MG09948.1 	PKNNMNASQLQNHFDAFYEDLWCELCKYGELEELVVCDNNNDH1LIGNVYARFKYEDSAQ
NCU03261.1	PKNRMNASQLQNHFDAFYEDIWCEMCKYGELEELVVCDNNNDH~LIGNVYARFKYEDSAQ
FG06860.1 	PKNRMNPSQLQNHFDAFYEDIWCELCKYGELEELVVCDNNNDH1LIGNVYARFKYEESAQ
AN9362.1  	PKSKMNPSQLQNHFDAFYEDVWCEMCKYGELEELVVCDNNNDH1LIGNVYARFKYEEDAQ
          	**..**.*************:***:****************** ************:.**

MG09948.1 	KACDELNSRWYAARPIYCELSPVTDFREACCRLNSGEGCTRGGFCNFIHRKNPSEELDRE
NCU03261.1	KACDDLNSRWYAARPIYCELSPVTDFREACCRLNSGEGCVRGGFCNFIHRKNPSPELERE
FG06860.1 	KACDELNSRWYAARPIYCELSPVTDFREACCRLNSGEGCVRGGFCNFIHRKNPSEELDRD
AN9362.1  	AACDALNSRWYAARPIYCELSPVTDFREACCRLNSGEGCVRGGFCNFIHRKDPSSELDRE
          	 *** **********************************.***********:** **:*:

MG09948.1 	LVLATKKWLKMRGRDERSVSRSPTPEPSRRRF
NCU03261.1	LELSTKKWLKMRPRS-RSPTRSPSPEPTRRRY
FG06860.1 	LTLSTKKWLKQRGRDERSASRSPTPEPTRRRY
AN9362.1  	LRLSTKKWLKERGRDARSVSRSPSPEPTRRRY
          	* *:****** * *. ** :***:***:***:
```
